# Heart Rate Variability and Decision-Making: Autonomic Responses in Making Decisions

**DOI:** 10.3390/brainsci11020243

**Published:** 2021-02-15

**Authors:** Giuseppe Forte, Matteo Morelli, Maria Casagrande

**Affiliations:** 1 Department of Psychology, Sapienza University of Rome, 00185 Rome, Italy; matteo.morelli92@gmail.com; 2 Department of Dynamic, Clinical Psychology and Health Studies, Sapienza University of Rome, 00185 Rome, Italy

**Keywords:** decision-making, HRV, autonomic nervous system, IGT, neurovisceral integration model, cognitive functions

## Abstract

Decision-making is one of the most crucial cognitive processes in daily life. An adaptable, rapid, and flexible decision requires integration between brain and body. Heart rate variability (HRV) indexes this brain–body connection and appears to be related to cognitive performance. However, its relationship with decision-making is poorly analyzed. This study investigates the relationship between HRV and the decision-making process, assessed through the Iowa Gambling Task (IGT). One hundred and thirty healthy university students (mean age = 23.35 ± 2.50) participated in the study. According to IGT performance, they were divided into high decision-makers (*n* = 79) and low decision-makers (*n* = 51). Heart rate variability was measured in the resting, reactivity (i.e., during IGT), and recovery phases. Higher vagally mediated HRV (vmHRV; indexed in frequency domain measures) was evidenced in good decision-makers in the resting, reactivity, and recovery phases. During the task, a higher vagal modulation after a first evaluation was highlighted in good decision-makers. In conclusion, HRV proves to be a valid index of inhibitory circuit functioning in the prefrontal cortex. The relationship with cognitive functions was also confirmed, considering the ability to inhibit disadvantageous responses and make better decisions.

## 1. Introduction

Decision-making is one of the most crucial cognitive processes in daily life [1,2]. It is necessarily required for important as well as irrelevant decisions or to make choices every day. Flexible decision-making supports easy decisions and oversees critical ones. Some of these choices are made based on clear and complete information, whereas others are made rationally [3,4]. However, unconscious processes play an important role in identifying the best option to follow. The necessity for enabling an adaptable, rapid, and flexible response to a perceived threat [5] requires integration between brain and body. Evidence of these interactions has been reported by different authors [5,6,7]. Specifically, the somatic marker hypothesis (SMH) [6,7] emphasizes the influence of the body response on behavior, highlighting that salient events automatically trigger changes in body states. The integration between visceral autonomic and perceptual information reinforces the representations of those stimuli, modeling behavioral responses. Autonomic responses seem to reflect learning the value of stimuli and the central feedback of body changes that can affect judgment [8].

Research has employed several physiological measurements to index the brain–body connection, such as heart rate variability (HRV) [9]. HRV reflects oscillations in the interval (ms) between consecutive heartbeats, which result mainly from parasympathetic (vagal) inputs to the heart via the sinoatrial node [10,11]. In the HRV spectrum, the high-frequency band (HF, from 0.15 to 0.40 Hz) was more often related to vagal-mediated influences [12]. Low HF-HRV could indicate a lack of the ability to respond to the changing environmental demands flexibly and generate appropriate responses and inhibit inappropriate ones. According to the neurovisceral integration model and polyvagal theory [12,13,14], HF-HRV is related to tonic inhibitory effects from the prefrontal cortex to subcortical structures, and its link with cognitive performance has largely been reported [15]. In this context, lower HF-HRV could indicate a dysregulation of the central autonomic network with greater arousal [13]. On the contrary, higher HF-HRV has been linked to more flexible strategies [16] and better performance on the executive level (for a review, see [15]). 

Functionally, HRV is associated with the activity of the ventromedial Prefrontal Cortex (vmPFC; for a review, see [17]). This area plays a fundamental role in the neurovisceral model, which considers it a center specialized in heart rate variability modulation. The somatic marker hypothesis suggests a central role of the vmPFC in modulating the ability to make decisions under uncertainty by coupling a specific event with the corresponding visceral state. 

This study aims to investigate the relationship between heart rate variability and the decision-making process, assessed through the Iowa Gambling Task (IGT) [18]. In particular, autonomic response while making a decision was analyzed in both high and low decision-makers.

First, we analyzed the resting state. Based on the neurovisceral model, HRV is supposed to be associated with IGT performance. According to the polyvagal theory and the neurovisceral integration model [12,13,14], since inhibitory control is reflected in higher HRV, we expected that the high decision-maker (high-DM) group would show higher resting HRV.

Furthermore, according to vagal tank theory [19], the relationship between HRV and decision-making during both reactivity and recovery phases was investigated. According to the somatic marker hypothesis [6,7] and vagal tank theory [19], a sympathetic vagal withdrawal response in the initial phases of the IGT (i.e., increase in HRV) is expected due to parasympathetic control, with a consequent decrease in HRV [20]. Specifically, better inhibition abilities are expected in high decision-makers than in low decision-makers. 

Regarding low decision-makers, a higher general sympathetic response (i.e., lower HRV) is expected.

## 2. Materials and Methods

### 2.1. Participants

Participants were recruited through university and internet advertisements. Participants with past or present psychiatric diagnoses and medical conditions and those who abused drugs or medications affecting HRV data were excluded (*n* = 5). The final sample included 130 healthy university students (mean age = 23.35 ± 2.50, range 18–30; 60.7% females).

In line with known procedures [21,22], the participants were divided into low and high decision-makers based on their IGT performance. According to Bull and colleagues [23], the proportion of net score was calculated for each participant by subtracting the proportion of choices from disadvantageous decks from that of advantageous decks. The formula used was as follows: ((C + D) − (A + B))/100. Individuals showing a proportion of less than 0 were assigned to the low-decision-maker group. Two groups were obtained: high decision-makers (high DM; *n* = 79; mean age = 23.2 ± 2.4; 46 females) and low decision-makers (low DM; *n* = 51; mean age = 23.5 ± 2.6; 33 females). 

### 2.2. Instruments and Measures

#### 2.2.1. Sociodemographic Interview

The face-to-face semi-structured anamnestic sociodemographic interview was adopted to obtain information about age, education, and daily habits, including alcohol consumption (number of drinks drunk per week), smoking (number of cigarettes smoked per day), and coffee use (number of cups drunk per day).

#### 2.2.2. Blood Pressure and Heart Rate

Blood pressure, systolic and diastolic, and heart rate were measured with an electronic device (Omron M3; HEM-7200-E, Omron Healthcare, Kyoto, Japan).

#### 2.2.3. Height and Weight

An Omron professional digital balance, calibrated in kg, was used to measure the weight. The height of each participant was measured using a wall-mounted anthropometer. These measures were used to calculate the body mass index (BMI) by dividing weight (in kilograms) by height (meters squared). 

#### 2.2.4. Heart Rate Variability

Electrocardiography (ECG) was performed using a Firstbeat Bodyguard 2 heart rate monitoring system (Firstbeat Analytics, Jyväskylä, Finland). The signal was detected by two Ag/AgCl electrodes (Ambu BlueSensor L, Ballerup, Denmark), one applied under the right collarbone and the other under the left rib cage. HRV was evaluated by calculating high frequency (HF-HRV; 0.15–0.40 Hz), which reflects changes in vagal control of the heart [24], and low frequency (LF-HRV; 0.04–0.15 Hz), which represents a mix between vagal and sympathetic activity [25,26] and is modulated by baroreflexes [27]. The signals were analyzed with Kubios software [28] (ver. 3.4.3., Kubios Oy, Kupio, Finland). For each participant, the following measures were calculated: resting, reactivity (i.e., during IGT task), and recovery. Following international guidelines [25,26], recovery and resting consisted of five-minute recordings, respectively, before and after the IGT performance. The IGT was divided into five blocks for each individual. This procedure allows for assessing the changes in the autonomic response during the test.

#### 2.2.5. Global Cognitive Functioning

Abstract reasoning performance was assessed with Raven’s Standard Progressive Matrices. Moreover, the Mini-Mental State Examination (MMSE [29]; Italian validation: [30]) was adopted to assess overall cognitive functioning. 

#### 2.2.6. Iowa Gambling Task (IGT)

Decision-making was assessed using the Iowa Gambling Task (IGT) [18]. This study adopted a digital version completely superimposable on the original analogical version [21].

##### Apparatus

The task was administered via E-Prime 2.1 software (Psychology Software Tools Inc., Pittsburgh, PA, USA) on a Lenovo computer, equipped with a 15-inch monitor and running the Windows 7 operating system.

##### Stimuli

The stimuli were four decks of cards (i.e., A, B, C, and D) presented on a green background. The back and front of the decks were the same (a red cover and a Joker of Spades, respectively) to reduce distraction and prevent the participants from using an unnecessary strategy guided by certain types of cards [31].

##### Procedure

Each card in the decks could lead to a win or a loss. However, the decks differed in the frequency and amount of wins and losses. Decks A and B were considered disadvantageous. They involved a larger short-term win ($100) but a long-term loss. Decks A and B differed in the frequency and magnitude of the loss. In deck A, the loss was more frequent but less plentiful than in deck B. With every 10 cards drawn for each deck, there was a loss of $1250 against a win of $1000. Overall, both decks A and B led to a loss of $250 for every 10 cards drawn. Decks C and D were more advantageous because they resulted in a smaller short-term payout ($50 each). Similar to decks A and B, these also differed in the frequency and magnitude of the loss. Deck C had more frequent losses, but of a lesser entity, than deck D. Every 10 cards drawn on these decks resulted in a win of $500 with a loss of $250. Therefore, they resulted in a win of $250 for every 10 cards selected. On the screen, the participants could see the amount of money they had won (written in green) and lost (written in red). The amount of money held was updated with each selection.

The task required the participants to select one card at a time from one of the decks. Each participant started with a $2000 credit, which could increase or decrease throughout the game. The participants were informed that some decks were more advantageous than others.

The participants’ response was made by pressing one of the four keys in the keyboard, corresponding to the deck they intended to choose. The test ended automatically after the individual made the hundredth selection. The participants were unaware of this rule. They also did not know when a loss would appear. The locations of the losses in this experiment were the same as those used by Bechara et al. [18].

### 2.3. General Procedure

**Figure 1:**
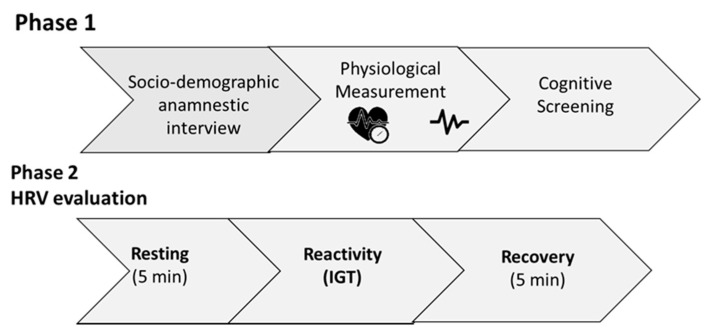
General procedure. HRV: Heart Rate Variability; IGT: Iowa Gambling Task.

Before the experiment, the participants received instructions they had to follow before the research started (i.e., do not smoke and do not drink coffee in the 2 h before the beginning of the test; do not drink alcohol 24 h before the test). The experiment was carried out at the laboratory of Health Psychology: Cognitive and Psychophysiological Assessment of the Department of Dynamic, Clinical Psychology and Health Studies of the Sapienza University of Rome. After the informed consent was signed, a semi-structured sociodemographic anamnestic interview was conducted. Then weight and height were recorded. Blood pressure was measured three consecutive times, according to guidelines provided by the Task Force for the management of arterial hypertension of the European Society of Cardiology (ESC) and the European Society of Hypertension (ESH) [32]. Then, the MMSE and Standard Progressive Matrices were administered. HRV in the resting and recovery phases was recorded for five minutes while the participants sat with knees at a 90° angle, both feet flat on the floor, hands on thighs, with palms facing upward and eyes closed, according to guidelines [25] and practical recommendations [25,26]. After the resting phase, the IGT was administered. HRV was recorded for the entire duration of the task. All participants were assessed individually. The whole procedure, lasting 40 min, took place in a quiet, dark, silent, and temperature-controlled room. Figure 1 shows a schema of the general procedure.

### 2.4. Data Analysis

To evaluate group differences in the demographics and clinical and cognitive variables, one-way analysis of variance (ANOVA), considering the group as a between variable, was computed on each of the following variables: age, education, smoking, coffee, BMI, systolic and diastolic blood pressure, HRV, Raven’s Standard Progressive Matrices, and MMSE. A chi-square comparison was used to test differences in sex distribution. If significant differences between the groups in BMI, age, and smoking were found, these variables were considered confounding variables and controlled in statistical analysis. The autonomic responses were evaluated by a 2 × 3 mixed ANOVA design that considered the group (high DM, low DM) as a between-subject variable, time (baseline, task, recovery) as a within-subject variable, and ln(HF-HRV) as a dependent variable. Finally, the HRV records of the IGT were divided into five phases corresponding to the different blocks of the task. These data were analyzed by a 2 × 5 mixed ANOVA design that considered the group (high DM, low DM) as a between-subject variable, the IGT block (1,2,3,4,5) as a within-subject variable, and ln(HF-HRV) as a dependent variable. Planned comparisons were conducted to analyze the interactions. For all the statistical analyses, the level of significance was accepted at *p* < 0.05. The statistical analyses were performed using the Statistica software (ver. 10.0, Dell, Round Rock, TX, USA).

## 3. Results

The demographics and clinical and cognitive variables of the two groups of participants are shown in Table 1.

The ANOVAs did not show significant differences between the groups for age, years of education, BMI, cups of coffee consumed daily, number of cigarettes smoked per day, systolic and diastolic blood pressure, and performance in the MMSE (all *F* < 1.1; all *p* > 0.29). However, the two groups differed significantly in heart rate (*F*_1,128_ = 6.9; *p* = 0.009; *η*^2^ = 0.05) and SPM (*F*_1,128_ = 7.99; *p* < 0.01; *η*^2^ = 0.07). Specifically, the high-DM group showed lower heart rate and higher abstract reasoning performance than the low-DM group.

### 3.1. Resting, Reactivity, and Recovery Differences

A significant main effect of the group (*F*_1,128_ = 6.63; *p* < 0.01; *η*^2^ = 0.04) emerged, with greater ln(HF-HRV) in the high-DM than the low-DM group (high DM: 6.31 ± 0.55 vs. low DM: 6.06 ± 0.54; see Table 2). Moreover, a significant main effect of condition (*F*_2,256_ = 7.54; *p* < 0.001; *η*^2^ = 0.04) revealed that the vagal tone was higher in the resting (*F*_1,128_ = 14.97; *p* < 0.001; *η*^2^ = 0.04; resting: 6.25 ± 0.52 vs. reactivity: 6.11 ± 0.55) and recovery (*F*_1,128_ = 4.31; *p* < 0.05; *η*^2^ = 0.02; recovery: 6.19 ± 0.51 vs. reactivity: 6.11 ± 0.55) conditions compared to the reactivity condition. No significant differences were found between resting and recovery (*F*_1,128_ = 3.17; *p* = 0.08). The group by condition interaction was not significant (*F*_2,246_ = 1.70; *p* = 0.18; *η*^2^ = 0.01). See Table 2.

### 3.2. Differences in IGT Blocks

**Figure 2:**
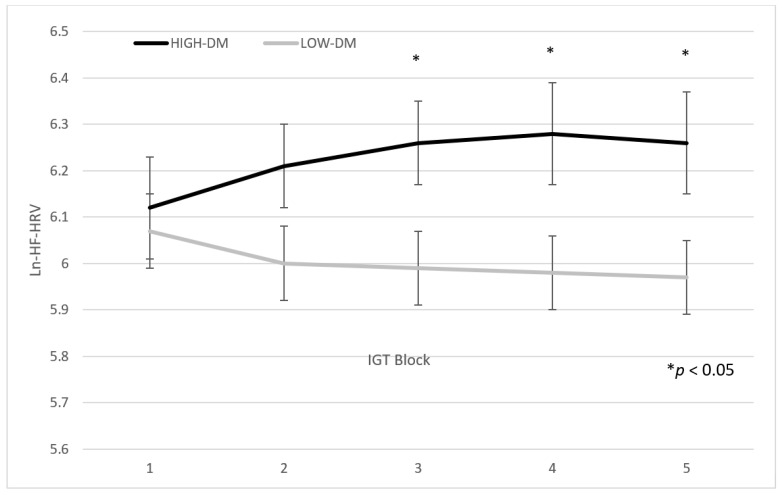
ln(HF-HRV) trend during Iowa Gambling Task of high and low decision-makers. HF: High Frequency; IGT: Iowa Gambling Task; DM: Decision makers.

A significant main effect of the group (*F*_1,128_ = 5.95; *p* < 0.01; *η*^2^ = 0.04) was confirmed (high DM: 6.31 ± 0.55 vs. low DM: 6.06 ± 0.54; see Table 2). Moreover, the group by block interaction was significant (*F*_4,512_ = 2.40; *p* = 0.04; *η*^2^ = 0.02). Planned comparisons showed that high decision-makers had higher ln(HF-HRV) than low decision-makers from third to fifth block (third block: *F*_1,128_ = 7.80; *p* = 0.006; *η*^2^ = 0; fourth block: *F*_1,128_ = 6.94; *p* = 0.009; *η*^2^ = 0.05; fifth block: *F*_1,128_ = 6.53; *p* = 0.01; *η*^2^ = 0.05), but not in the first two blocks (first block: *F*_1,128_ < 1; *p* = 0.57; *η*^2^ = 0.002; second block: *F*_1,128_ = 3.34; *p* = 0.07; *η*^2^ = 0.001) (see Figure 1). Table 2 and Figure 1 show the means ± standard deviations of the two groups’ performance in the five blocks of the IGT. See Table 3 and Figure 2.

## 4. Discussion

There is growing evidence showing that the autonomic nervous system can influence cognitive performance (for a review, see [15]), confirming the neurovisceral integration hypothesis regarding the association between HRV and both executive and non-executive functions [12,14]. Our results extend the evidence of previous studies, underlining that higher vagally mediated HRV (vmHRV) is also associated with decision-making. According to previous studies (for a review, see [15]), and with the assumption of the neurovisceral integration model and polyvagal theory [5,12,14], higher resting-state vmHRV (indexed in frequency domain measures), which reflects better functioning of inhibitory circuits, was confirmed to be related to cognitive performance in good decision-makers (i.e., better performance in IGT). These results are not influenced by other confounding variables (e.g., gender, BMI, blood pressure, smoking habits).

The Iowa Gambling Task measures the ability to make decisions in uncertain situations in which people have limited knowledge about contingencies and are strictly guided by rewards and punishments [6]. Previous studies underline the association of IGT performance with HRV, considering the time and frequency domains of HRV [33,34,35]. According to Bechara and Damasio [7,36], the somatic marker hypothesis about neural substrates, including the medial prefrontal cortex (mPFC), amygdala, insular cortex, somatosensory cortex, brainstem nuclei, anterior cingulate cortex (ACC), and dorsolateral prefrontal cortex (DLPFC), is directly associated with performance in IGT. The ACC and DLPFC are also parts of the parasympathetic suppression network via which the PFC might control vmHRV [37], providing a neurofunctional explanation of our results. Low HF-HRV indicates decreased prefrontal inhibitory influences on subcortical structures and consequently a reduced control of executive, attentional, and self-regulation processes [14,38]. This functioning could explain the association between vmHRV and IGT performance. Moreover, our findings confirmed the role of the autonomic nervous system in cognitive functioning, supported by previous studies [16,39,40,41,42]. Due to higher emotional control, which allows being guided not merely by punishments and rewards but also by long-term consequences, high decision-makers perform better in the Iowa Gambling Task.

Another aim of our study was to define an autonomic pattern associated with the decision-making process. To the best of our knowledge, this is the first study explicitly addressing the relationship between the IGT and HF-HRV, considering autonomic change during the task to define an autonomic pattern of the good decision-maker. Results considering IGT blocks highlight that the difference between high and low decision-makers in vmHRV was evidenced only in the last three blocks. These findings clearly show a trend of HF-HRV during the IGT. In the first block (i.e., the first 20 cards), no difference was found between the groups, and high decision-makers showed an HRV decrease similar to low decision-makers due to the participants’ involvement in the task. This pattern was partially confirmed in the second block. However, starting from the third block, high decision-makers presented an increase in HF, which probably represents an adaptation to the task [8]. This pattern, confirmed until the end of the task, supports the hypothesis that basal Autonomic Nervous System (ANS) activity is critical in the evolution of decision-making processes. Better inhibitory control of PFC regarding making a decision starts after the initial acquisition of knowledge. The prefrontal cortex is considered to have a primordial role in the approach-related emotional–motivational system, which mediates and facilitates appetitive behaviors [43]. Furthermore, the prefrontal cortex inhibits limbic regions such as the amygdala, a key structure in the withdrawal-related emotional system that facilitates withdrawal from aversive stimulation sources [43]. According to the somatic marker hypothesis of decision-making [44], increased prefrontal activity leads to diminished amygdala activity and lower emotional reactivity (i.e., lower somatic marker inhibitory influences on decision-making). Thus, autonomic activity and reactivity appear to have an important role in the decision-making process.

Finally, in line with the vagal tank theory (VTT) [19], this study shows vagal modulation during a cognitive task. During IGT performance, vmHRV seems to decrease compared to resting and returns to normal in the recovery condition. This physiological response, probably due to exposition to a new challenge, according to VTT, is considered an adaptive concern. Indeed, in our sample, both groups exhibit this physiological response.

### Limitations and Future Perspectives

The main limitation of this study is the sample size, which may have compromised statistical power. Second, although there are no differences between the groups, the confounding variables (such as BMI) should be better controlled. Third, the sample is composed of young participants, and the results cannot be generalized for other age ranges, such as the elderly. However, it would be interesting to deepen this aspect because cardiac vagal activity tends to decline with age [10], and the nature of the relationship with decision-making can change over time. Another limitation could be due to the use of MMSE to assess global cognitive functioning. MMSE is certainly more suitable for assessing overall cognitive functioning in the elderly population with mild cognitive impairment or dementia (e.g., Alzheimer’s disease, see [45,46]), but global cognitive performance in young people should be evaluated with a more complex test battery. Moreover, HRV could be measured for more time to assess autonomic aspects better. Although previous results found higher reliability using low-duration HF-HRV measures [47], the extent of each IGT block is lower than the five minutes gold standard term. This could have influenced the analysis, and further studies need to clarify these aspects. Finally, according to a previous study (for a review, see [15]) and given the higher correlation with root mean square successive difference (RMSSD), we evaluate only HF-HRV; further studies are needed to assess the possible difference between time and frequency domain measures. Given these limitations, the present results should be treated with caution and considered preliminary.

Despite some limitations, our results may have practical applications, considering the effects of interventions aimed at increasing HRV (e.g., physical exercise, biofeedback, drugs, transcutaneous vagus nerve stimulation) and preventing or reducing cognitive impairment associated with executive function decline [45,46]. Further studies are needed to replicate and generalize the results obtained. For example, it could be useful to consider other decision-making tasks that are focalized on different aspects of this construct, such as the Balloon Analogue Risk Task, dilemmas, or the Game of Dice Task. Another possible future direction is to investigate the HRV response by administering other IGT versions, for example, characterized by more than 100 cards to define the autonomic pattern better. A similar choice could be useful because the task duration allows more accurate autonomic recording over a longer time. Moreover, the use of more than 100 cards has been associated with better performance in the test [48]. It could be interesting to evaluate this aspect in the clinical population related to lower cognitive functioning, such as hypertension [49,50] and obesity [51]. Future research may investigate social decision-making.

## 5. Conclusions

To the best of our knowledge, this is the first study to focus attention on good decision-makers to define an autonomic pattern. A relationship between vmHRV and cognitive functioning was confirmed, specifically in decision-making. Our findings highlight an autonomic pattern characterized by a vagal modulation that starts after a first evaluation of the task. Good decision-makers had a higher vagal tone in the resting state and during the task. Not influenced merely by options more attractive at the moment but disadvantageous in the long term, they appeared to be more capable of making choices guided by the future consequences of their actions. Accordingly, participants with higher vmHRV seemed more susceptible to the further consequences of their actions, while lower HRV was associated with a sort of myopia for the future. A high HRV is associated with better performance in executive functions [15], with more efficient emotional regulation [52], with better emotional recognition [53], and with social engagement [54]. By contrast, a low HRV is associated with a series of complications such as psychopathology [55], hypertension [56], diabetes [57], and higher mortality following heart failure [58]. This study shows that greater HRV can be used as a marker of better decision-making skills and suggests using some techniques to increase vagal tone and improve an individual’s ability to make decisions in uncertain conditions. In conclusion, HRV proves to be a valid index of a person’s ability to self-control, emotionally regulate himself, inhibit disadvantageous responses, and make better decisions.

## Figures and Tables

**Table 1:** Means (M) and standard deviations (SD) of the demographics and clinical and cognitive characteristics of the two groups of participants.

	High DM (*n* = 79)	Low DM (*n* = 51)	*F/X* ^2^	*p*	*η* ^2^
Sex					
Male	33	18	<1	0.46	
Female	46	33			
Age	23.2 (2.4)	23.5 (2.6)	<1	0.49	0.004
Years of education	16.4 (1.8)	16.1 (1.8)	<1	0.33	0.007
BMI	22.4 (3.2)	22.6 (3.5)	<1	0.75	0.0001
SBP	116.7 (10.9)	116.9 (11.1)	<1	0.93	0.0001
DBP	71.1 (7.6)	72.5 (8.2)	1.02	0.31	0.008
HR	74.3 (11.3)	80.3 (13.9)	6.9	0.009	0.05
Smoke (cigarettes per die)	3.3 (4.9)	2.8 (5.4)	<1	0.54	0.003
Coffee (per die)	1.8 (1.5)	1.7 (1.5)	<1	0.67	0.001
MMSE score	29.7 (0.5)	29.6 (0.7)	1.1	0.29	0.009
SPM score	49.16 (5.8)	45.2 (8.30)	7.99	0.006	0.07
IOWA score	0.20 (0.22)	−0.16 (0.13)	103.52	<0.001	0.44

BMI: body mass index; SBP: systolic blood pressure; DBP: diastolic blood pressure; HR: heart rate; MMSE: Mini-Mental State Examination; SPM: standard progressive matrices; IOWA: Iowa Gambling Task.

**Table 2:** Means (M) and standard deviations (SD) of ln(HF-HRV) in decision-making (DM) groups.

	High DM (*n* = 79)	Low DM (*n* = 51)	*F*	*p*	*η* ^2^
ln(HF baseline)	6.40 (0.55)	6.10 (0.54)	9.50	0.003	0.06
ln(HF total IOWA)	6.23 (0.61)	6.00 (0.63)	3.75	0.04	0.02
ln(HF recovery)	6.30 (0.58)	6.08 (0.56)	4.61	0.03	0.03

ln: Natural logarithm; HF: high-frequency; IGT: Iowa Gambling Task.

**Table 3:** Means (M) and standard deviations (SD) of ln(HRV) in decision-making (DM) groups.

	High DM (*n* = 79)	Low DM (*n* = 51)	*F*	*p*	*η* ^2^
ln(HF-HRV) IGT first block	6.12 (0.56)	6.07 (0.71)	<1	0.73	0.001
ln(HF-HRV) IGT second block	6.21 (0.55)	6.00 (0.70)	3.34	0.07	0.02
ln(HF-HRV) IGT third block	6.26 (0.46)	5.99 (0.63)	7.80	0.006	0.05
ln(HF-HRV) IGT fourth block	6.28 (0.59)	5.98 (0.69)	6.94	0.009	0.05
ln(HF-HRV) IGT fifth block	6.26 (0.56)	5.97 (0.70)	6.53	0.01	0.05

## Data Availability

Data available on request.
